# Neuroendocrine control of glucose homeostasis: integrative mechanisms from the hypothalamus to the brainstem

**DOI:** 10.3389/fendo.2025.1731725

**Published:** 2025-12-02

**Authors:** Nilda Gallardo, Sara Artigas-Jerónimo, Lorena Mazuecos, Antonio Andrés

**Affiliations:** 1 Biochemistry Section, Faculty of Sciences and Chemical Technologies, University of Castilla-La Mancha, Ciudad Real, Spain; 2 DOE Research Group, Institute of Biomedicine, IDISCAM, Ciudad Real, Spain

**Keywords:** hypothalamus, brainstem, glucose homeostasis, central regulatory circuits, vagus nerve, type 2 diabetes

## Abstract

Glucose is vital for brain physiological function, acting as the primary energy source that supports cognitive processes, neurotransmitter production, and overall health. The brain requires a constant supply of glucose, and the body has evolved protective mechanisms to maintain this supply during hypoglycemia. Increased appetite and food intake is a fundamental protective response. The precise network of brain regions, nerves, and connections responsible for initiating and coordinating these responses has not been fully identified or mapped. Neuroendocrine centers within the hypothalamus and brainstem monitor metabolic signals such as glucose, insulin, and leptin to regulate autonomic outflow, endocrine function, and behavior. Disruption of these central regulatory circuits contributes significantly to the pathogenesis of metabolic disorders, including obesity and type 2 diabetes mellitus (T2DM). Interestingly, incretin-based pharmacotherapies and bariatric surgery suppress food intake by acting on the brain, thereby enhancing the regulation of glucose homeostasis. This review summarizes current knowledge on the neural and hormonal pathways, including incretin signaling, involved in physiological glucose regulation, the mechanisms underlying their dysfunction in disease states, and the recent advances pointing to potential central targets for therapeutic intervention.

## 1 Introduction

Glucose is the primary energy substrate for most tissues and the almost exclusive fuel for the brain in both rodent and human. As noted by Mergenthaler et al. (2013) (1), the mammalian brain depends on glucose to sustain neuronal activity, neurotransmitter synthesis, and overall cognitive function. Thus, precise control of glucose metabolism is essential for brain and systemic health. To ensure a constant energy supply, plasma glucose concentrations are tightly regulated through highly coordinated mechanisms, a process known as glucose homeostasis (1, 2).

Traditionally, glucose homeostasis has been attributed to the balance between insulin and glucagon secretion by the pancreas (2). However, growing evidence highlights a pivotal role of the central nervous system (CNS) in sensing metabolic status and actively orchestrating systemic glucose regulation, shifting the paradigm from a passive to an integrative control system (3, 4). Key determinants of glucose homeostasis, such as insulin and glucagon secretion, peripheral insulin sensitivity, and glucose utilization, are under central modulation by the brain.

Within the brain, the hypothalamus and brainstem act as neuroendocrine hubs that integrate hormonal, nutritional, and neural signals to regulate feeding, autonomic tone, and endocrine outputs. Specialized hypothalamic glucose-sensing neurons detect changes in glucose availability through mechanisms shared with pancreatic β-cells and translate this information into adaptive autonomic and behavioral responses to maintain glucose stability. Through reciprocal connections with other brain regions, the hypothalamus and brainstem coordinate food intake, thermogenesis, and systemic energy balance.

Disruption of these regulatory circuits is increasingly recognized as a key contributor to metabolic disease. Central insulin and leptin resistance, hypothalamic inflammation, and impaired brainstem autonomic control are implicated in the pathogenesis of obesity and T2DM (4). Importantly, these alterations often precede overt hyperglycemia, suggesting that neuroendocrine dysfunction constitutes an early, causal step in metabolic deterioration (5) and may accelerate brain aging (6, 7) in both rodents and humans. Diet-induced hypothalamic stress, particularly from high-fat diets, further amplifies inflammatory and oxidative pathways, exacerbating both metabolic and cognitive decline.

Understanding how the hypothalamus and brainstem sense and integrate metabolic signals is therefore essential for elucidating the mechanisms underlying metabolic disease and identifying new therapeutic strategies. In this review, we summarize current insights into the neuroendocrine control of glucose homeostasis, discuss mechanisms of central dysfunction in metabolic disease, and highlight emerging therapies targeting brain pathways to restore systemic glucose balance. Much of the available evidence comes from rodent studies, as functional validation in humans remains limited.

## 2 Homeostatic control of glucose levels by the CNS

### 2.1 Hypothalamic regulation of glucose homeostasis

The core mechanisms of hypothalamic regulation of glucose homeostasis are conserved across mammals. The hypothalamus, particularly the arcuate nucleus (ARC), ventromedial (VMH), dorsomedial (DMH), and paraventricular (PVN) nuclei, plays a central role in regulating energy balance by sensing energy stores, metabolic signals, and nutrient availability. Adiposity signals such as insulin and leptin convey information about long-term energy sufficiency (8, 9), whereas gut-derived hormones including ghrelin, peptide YY (PYY), cholecystokinin (CKK), gastric inhibitory polypeptide (GIP), and glucagon-like peptides (GLP-1/GLP-2) reflect short-term changes in nutrient status and dietary intake across the life course (10–13).

Moreover, circulating nutrients (glucose, free fatty acids) and metabolites provide additional cues reflecting shifts in metabolic and environmental conditions. In addition, the biological clock within the hypothalamus also influences daily rhythms of glucose metabolism, with disrupted clock gene expression being closely associated with insulin resistance and type 2 diabetes (14).

Distinct hypothalamic neuronal populations in the ARC sense these signals and coordinate autonomic and neuroendocrine outputs to regulate feeding, energy expenditure, and glucose metabolism (4, 15). The proopiomelanocortin (POMC) and agouti-related peptide/neuropeptide Y (AgRP/NPY) neurons are two well-characterized populations that exert opposing influences on energy and glucose homeostasis. POMC neurons are anorexigenic and glucose-excited, promoting insulin sensitivity and glucose uptake when glucose and nutrients are abundant (16, 17). Conversely, AgRP/NPY neurons are orexigenic and glucose-inhibited; they become more active when blood glucose levels are low and ghrelin is high, stimulating hepatic glucose production to restore normoglycemia (18, 19).

Elegant studies using optogenetic and chemogenetic tools have confirmed, in mice, the dominant role of AgRP/NPY neurons in driving feeding behavior and acute metabolic adaptations during fasting and hypoglycemia (18, 20). In contrast, hypothalamic POMC neurons exert slower but more sustained effects, particularly in modulating sympathetic tone and energy expenditure (17). Nevertheless, the POMC-Melanocortin 4 Receptor (MC4R) circuit is essential for the body’s ability to counteract hypoglycemia, as demonstrated by studies showing that POMC or MC4R deficiency impairs glucagon secretion and hepatic glucose production in diabetic mice (21).

Building on this framework, recent evidence indicates that the functional interplay between orexigenic AgRP/NPY and anorexigenic POMC neurons extends beyond appetite control to encompass systemic glucose regulation. According to De Solis et al., simultaneous activation of AgRP/NPY neurons and inhibition of POMC neurons exert additive effects on feeding behavior yet produce distinct outcomes on systemic insulin sensitivity and hepatic gluconeogenesis compared with their independent modulation (22). Notably, this cooperative effect on feeding is not fully maintained in female mice. These observations suggest that hypothalamic circuits controlling energy balance operate through sex-dependent coordination between orexigenic and anorexigenic neurons. Such differences may contribute to the sexually dimorphic regulation of glucose homeostasis and the variable susceptibility to metabolic disorders observed across sexes.

Moreover, AgRP/NPY and POMC neurons in the ARC project to the VMH and DMH hypothalamic nuclei, as well as to second-order neurons in the PVN and extra-hypothalamic regions such as the brainstem. Through these connections, they influence both autonomic outflow and behavior, thereby coordinating adaptive responses in feeding, energy expenditure, and glucose homeostasis (20, 22).

The VMH and PVN contain specialized neurons that sense glucose, fatty acids, insulin, and leptin, integrating intrinsic detection with peripheral signals to monitor whole-body energy status. Within the VMH, glucose-excited (GE) neurons respond to postprandial hyperglycemia, while glucose-inhibited (GI) neurons detect hypoglycemia and initiate counterregulatory responses. Their activity is governed by mitochondrial ATP production and redox state, emphasizing the critical role of cellular energy metabolism in hypothalamic glucose sensing (23, 24).

Ultimately, PVN neurons modulate sympathetic and parasympathetic activity to regulate hepatic glucose production, glycogen storage, and systemic glucose availability, acting as a key effector that connects hypothalamic sensing to peripheral glucose homeostasis (25). Hypothalamic nuclei, including the PVH, LH, and ARC, connect extensively with brainstem and spinal autonomic centers, such as the nucleus of the solitary tract (NTS), dorsal motor nucleus of the vagus (DMV) and the intermediolateral nucleus of the spinal cord (IML), which relay hypothalamic signals to pancreatic islets, liver, and other metabolic tissues. Through these circuits, the hypothalamus regulates sympathetic and parasympathetic outputs, controlling pancreatic function, adipose storage, thermogenesis, and overall glucose homeostasis thereby closing the loop between central sensing and peripheral control of glucose.

### 2.2 Central signaling: insulin and leptin crosstalk

Insulin and leptin play a crucial role in the central regulation of glucose homeostasis, by informing the brain of the energy status of the body. In adulthood, both hormones act on hypothalamic neurons that control food intake, energy expenditure and glucose metabolism via shared intracellular pathways, including PI3K–Akt and STAT3 signaling cascades. These pathways suppress hepatic glucose production, enhance peripheral insulin sensitivity, and regulate food intake. Disruption of insulin–leptin crosstalk contributes to central insulin and leptin resistance, a hallmark of obesity and type 2 diabetes (15, 26).

In addition, leptin activates hypothalamic circuits that regulate glucose homeostasis via the autonomic nervous system, modulating pancreatic insulin and glucagon secretion and influencing liver, brown adipose tissue, and skeletal muscle metabolism. Remarkably, leptin signaling in POMC neurons can normalize glycemia, increase physical activity, and prevent diabetes, independently of food intake or body weight (17). Importantly, leptin can also improve glucose control even under insulin-deficient conditions (27).

Together, insulin and leptin act as complementary neuroendocrine signals that coordinate central control of energy balance and glucose metabolism, highlighting their synergistic role in maintaining systemic metabolic homeostasis. Consistent with animal studies, human and translational data indicate that perinatal perturbations in leptin and insulin signaling can reprogramming the hypothalamic control of glucose metabolism (28).

### 2.3 Developmental programming of the hypothalamus and its early-life sensitivity

Beyond their role in adulthood, AgRP and POMC neurons undergo a critical developmental window that shapes lifelong metabolic regulation, including the regulation of glucose homeostasis. This period is regulated by perineuronal nets (PNNs), specialized extracellular matrix structures that envelop leptin receptor-positive GABAergic neurons within the ARC, limiting plasticity but ensuring the stability of this inhibitory neuronal circuit throughout adulthood in humans and rodents (29). Although the timing of hypothalamic maturation differs between species, postnatal in rodents *versus* prenatal in humans, the concept of a developmentally restricted period of metabolic sensitivity appears conserved.

During this period, leptin acts as a neurotrophic factor, promoting the outgrowth of AgRP projections from the ARC to downstream targets, including the PVN and DMH (30, 31). This neurotrophic effect is time-limited: in leptin-deficient (*ob/ob*) mice, neonatal leptin replacement can largely restore AgRP projections, whereas treatment after postnatal day 28 fails to rescue circuit development. Moreover, the recovery of PVN projections is more robustly in females than in males. All of these indicate that leptin’s trophic effects are restricted to the developmental critical period for neuronal maturation (29, 32).

**Figure 1:**
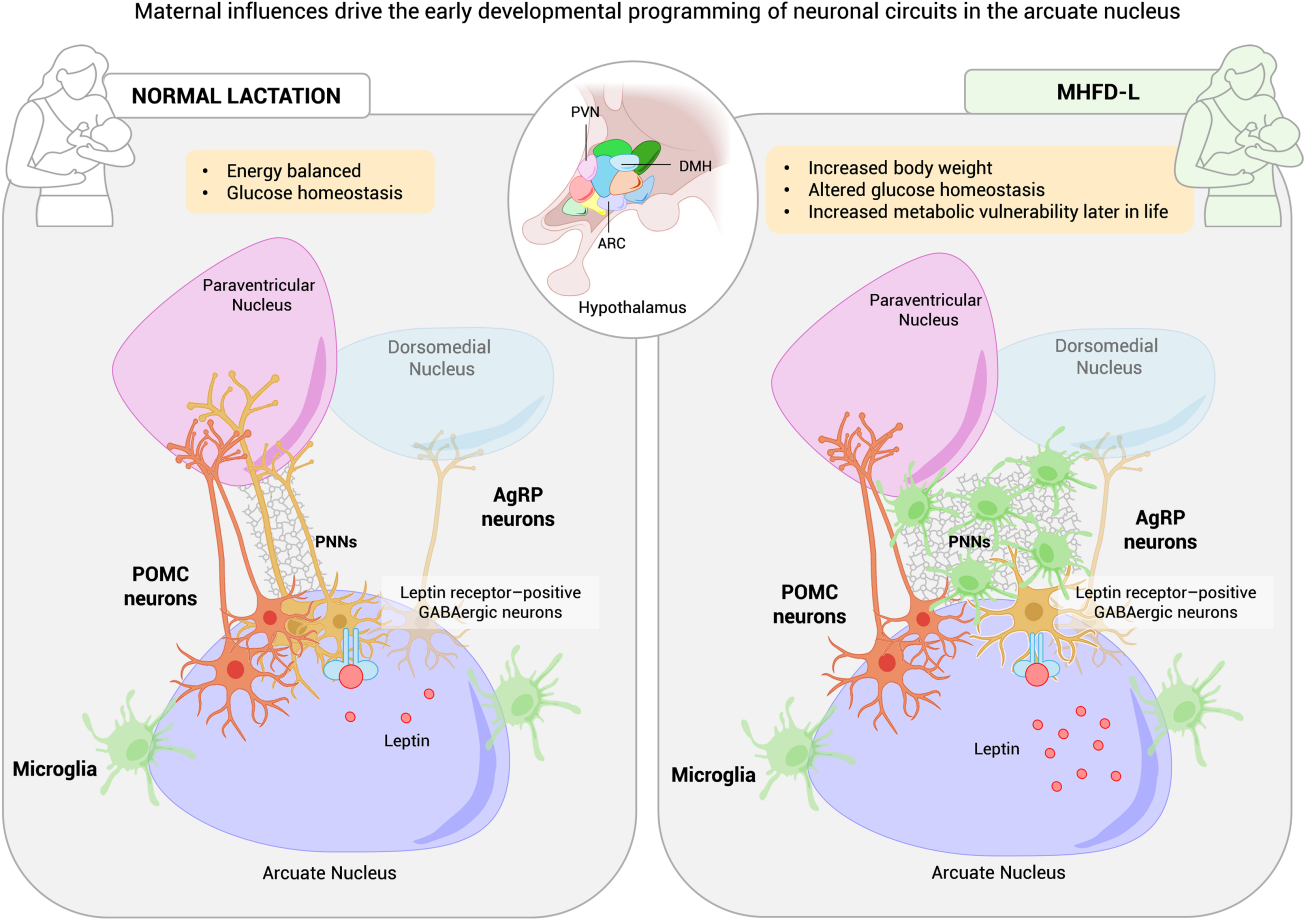
Impact of maternal high-fat diet on perineuronal nets (PNNs) and hypothalamic circuit plasticity controlling body weight and glucose metabolism. Schematic representation of how maternal exposure to a high-fat diet (HFD) alters the formation and remodeling of perineuronal nets (PNNs) in the arcuate nucleus (ARC) of offspring during the critical period of neuronal maturation. In both humans and rodents, maternal HFD during lactation (MHFD-L) increases perinatal leptin levels, disrupts PNN organization, and activates microglia, leading to degradation of AgRP neuronal terminals in the paraventricular nucleus (PVN). The resulting reduction in PVN-directed AgRP projections may predispose offspring to excessive weight gain and increased susceptibility to obesity and glucose dysregulation later in life. MHFD-L, maternal high-fat diet during lactation; Violet, ARC; Rose, PVN; Blue, DMH; Green, microglia; Orange, POMC neurons; Yellow, AgRP/NPY neurons.

PNNs are also influenced by dietary interventions. Maternal high-fat diet during lactation (MHFD-L) disrupts PNNs maturation and enhances microglial activity in hypothalamic nuclei, leading to active engulfment of AgRP terminals within the PVN. This microglial activity reduces AgRP innervation density and remodels hypothalamic circuits, programming higher body weight and increased susceptibility to obesity and glucose dysregulation later in life (33, 34). Notably, microglial depletion during this postnatal period prevents both the reduction in PVN AgRP projections and the excessive weight gain observed in MHFD-L offspring, underscoring microglia as critical mediators of this developmental programming (29) (**Figure 1**).

Nutritional environment, and microglial activity converge to shape hypothalamic melanocortin circuits, with enduring effects on feeding behavior, energy expenditure, and glucose homeostasis. This developmental plasticity represents both a window of vulnerability, where adverse nutritional or hormonal cues can program future metabolic disease by impairing hypothalamic responses to insulin and leptin (33, 35), and a window of opportunity for intervention. Notably, this vulnerability appears to be more pronounced in human males. Although it remains unclear how PNNs themselves modulate blood glucose levels, establishing timely strategies aimed at normalizing circuit development during neuronal maturation could reduce the lifelong risk of obesity and type 2 diabetes.

### 2.4 Incretin mimetics in the central control of glycemia

Incretin mimetics such as GLP-1 receptor agonists (GLP-1RAs) are highly effective antidiabetic agents that enhance insulin secretion, improve glycemic control, and promote sustained weight loss. Recent findings show that both GLP-1R agonists, such as semaglutide, and bariatric surgery converge on central circuits controlling energy balance, although the neural mechanisms of surgery remain less defined. Beyond their potent anorectic actions, these interventions activate vagal afferents, brainstem nuclei like the NTS, and hypothalamic melanocortin pathways to modulate food intake and glucose homeostasis (36).

GLP-1 receptors are broadly distributed in the hypothalamus, brainstem, and mesolimbic system, where they exert region-specific effects on satiety, reward, and glycemic regulation. Preproglucagon (PPG) neurons in the NTS represent the main source of endogenous GLP-1; their activation suppresses feeding and modulates autonomic output. However, pharmacological GLP-1RAs act partly independently of these neurons, indicating that endogenous and therapeutic GLP-1 engage distinct yet overlapping gut-brain pathways (37, 38).

Recent studies identified glucose-sensing GLP-1R neurons in the DMH that lower blood glucose by enhancing vagal output to the pancreas through cAMP–PKA signaling (39). Optogenetic and imaging approaches further show that GLP-1R-expressing neurons in the ARC, PVN, and DMH nuclei inhibit AgRP/NPY neurons, providing a direct mechanism through which central GLP-1 signaling reduces hunger and improves glycemic control (40, 41). Together, these findings delineate a hierarchical GLP-1 network that integrates peripheral and central inputs to coordinate satiation, energy expenditure, and autonomic output.

Neuroimaging and preclinical data support a model where endogenous and pharmacological GLP-1 actions overlap but are not identical, involving parallel gut-brain loops. This dual signaling framework explains the remarkable efficacy of GLP-1RAs in producing durable metabolic improvements across species (42).

Building on these mechanisms, dual and triple incretin receptor agonists represent a major advance in metabolic therapy. Dual GLP-1/GIP agonists such as tirzepatide, and triple agonists targeting GLP-1, GIP, and glucagon receptors such as retatrutide, integrate complementary pathways to optimize glucose control, enhance fat oxidation and thermogenesis, and improve cardiovascular outcomes (43). These next-generation incretin agonists harness synergistic central and peripheral effects to achieve superior glycemic and weight benefits compared to single GLP-1 agonists.

Importantly, evidence from human and preclinical studies indicates sex-dependent differences in incretin responsiveness. Enhanced GLP-1R expression in female brain regions involved in aversive and reward processing, along with estrogen-dependent modulation of GLP-1 signaling, may underlie greater treatment efficacy and tolerability in females (40). These findings highlight the need to integrate sex as a biological variable in future research.

Altogether, the emerging picture places GLP-1 not only as a gut-derived hormone but as a central neuropeptide coordinating satiety, autonomic regulation, and glucose homeostasis. Understanding how endogenous and pharmacological incretin pathways interact, across neural circuits, metabolic organs, and sex-specific contexts, will be crucial to developing the next generation of personalized anti-obesity and antidiabetic therapies.

## 3 Brainstem and autonomic regulation of glucose homeostasis: insights into the gut–brain-liver axis

**Figure 2:**
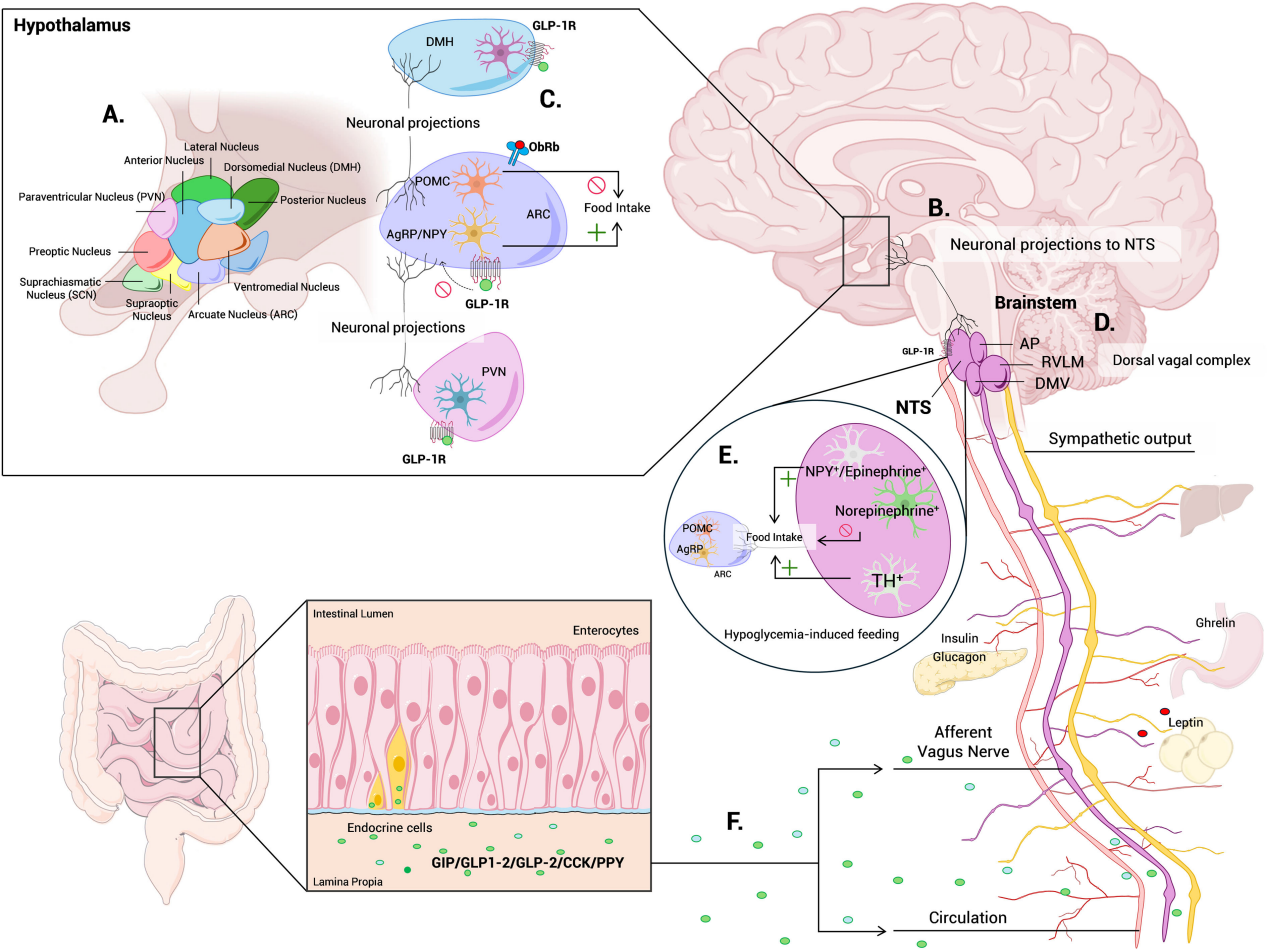
Neuroendocrine networks integrating hypothalamic and brainstem mechanisms of glucose homeostasis. Schematic overview summarizing the central circuits that coordinate glucose homeostasis through the hypothalamus-brainstem axis. **(A)** The ARC, DMH, VMH, LH, and PVN integrate hormonal and nutrient-derived signals via AgRP/NPY, POMC, and glucose-sensing neurons to regulate energy balance. **(B)** PVN projections to the NTS modulate autonomic activity controlling hepatic glucose output, glycogen storage and systemic glucose availability. **(C)** GLP-1 receptor-expressing neurons in the DMH and brainstem enhance hypothalamic-brainstem communication, promoting satiation and glucose control. **(D)** NTS, DMV, AP and RVLM areas in the brainstem provides essential autonomic control that maintains glucose homeostasis. **(E)** An ascending brainstem-hypothalamus pathway selectively drives appetite under energy deficit and hunger cues, in addition to its classical satiety function. **(F)** The gut-liver-brain axis contributes via vagal afferents, gut peptides (GLP-1, GIP, CCK, PYY), and microbiota-derived metabolites to modulate enteroendocrine signaling and neural pathways. ARC, arcuate nucleus; DMH; dorsomedial nucleus of hypothalamus; VMH; ventromedial nucleus of hypothalamus; LH, lateral hypothalamic area; PVN, paraventricular nucleus of hypothalamus; AgRP/NPY, agouti related peptide/neuropeptide Y neurons; POMC, pro-opiomelanocortin neurons; NTS, nucleus tractus solitaries; DMV, dorsal motor nucleus of the vagus; AP, area postrema; RVLM, rostral ventrolateral medulla; GLP-1, glucagon-like peptide 1; GIP, gastric inhibitory polypeptide; CCK, cholecystokinin; PYY, peptide YY; Violet, ARC; Rose, PVN; Blue, DMH; Green, microglia; Orange, POMC neurons; Yellow, AgRP/NPY neurons.

While the hypothalamus is a central hub for energy balance and glucose regulation, the brainstem provides essential autonomic control that maintains glucose homeostasis within narrow physiological limits (44, 45). Key regions in the brainstem include the dorsal vagal complex (DVC), comprising the nucleus of the solitary tract (NTS), dorsal motor nucleus of the vagus (DMV), and area postrema (AP), as well as the rostral ventrolateral medulla (RVLM), which together integrate visceral and hormonal signals and coordinate autonomic outputs to regulate pancreatic hormone secretion, hepatic glucose production, and peripheral glucose utilization (**Figure 2**).

The vagus nerve contains the primary sensory neurons that monitor the gastrointestinal environment and transmit this information to the brainstem. The NTS serves as a major integrative center for visceral afferents from the gastrointestinal tract, liver, and pancreas. Vagal and sympathetic afferents from the gut synapse in the NTS, where neuronal activity modulates insulin secretion and hepatic glucose output (46, 47).

Recent advances have refined our understanding of gut-brain communication as a central regulator of metabolic homeostasis. Using intersectional genetic approaches, Borgmann et al., demonstrated that distinct populations of gut-innervating vagal afferents exert differential control over food intake and glucose metabolism by engaging separate neural circuits in the brain (48). Consistently, activation of intestinal mechanoreceptors during feeding generates satiety signals transmitted via vagal afferents to the nucleus tractus solitarius (NTS), where they inhibit hypothalamic AgRP/NPY neurons and activate anorexigenic pathways (49). By modulating autonomic balance-reducing sympathetic drive and enhancing parasympathetic signaling-gut-derived sensory inputs lower hepatic glucose output and improve insulin sensitivity. These findings highlight vagal sensory pathways as key integrators of intestinal, hepatic, and hypothalamic signals in the neurohormonal control of glucose homeostasis (**Figure 2**).

Within this framework, catecholaminergic circuits in the NTS integrate visceral afferent inputs to adapt feeding and glucose metabolism to changing energy states. Epinephrine/NPY-expressing neurons promote feeding, norepinephrine neurons suppress it, and a subset of tyrosine hydroxylase–expressing neurons drives glucoprivic feeding through hypothalamic AgRP and POMC neurons, revealing an orexigenic role for the vagal-NTS circuit beyond its classical satiety function (50–53) (**Figure 2**).

The NTS also receives descending projections from hypothalamic PVN, LH, and ARC neurons, allowing bidirectional communication that integrates circulating signals such as glucose, ghrelin, GLP-1, and leptin to coordinate autonomic and neuroendocrine regulation. Parallel brainstem centers, including the RVLM and the area postrema, contribute to sympathetic outflow that promotes hepatic gluconeogenesis, inhibits insulin secretion, and regulates feeding and lipolysis. Collectively, hypothalamic and brainstem neurons, together with sympathetic preganglionic circuits, form an integrated network that coordinates feeding behavior and glucose balance, whose dysfunction contributes to impaired hypoglycemia awareness, hyperglycemia in type 2 diabetes, and hormonal resistance (54–60).

The gut microbiota interfaces with the gut-liver-brain axis through bidirectional communication encompassing neural, immune, and endocrine pathways. This network involves vagal signaling, microbial metabolites such as short-chain fatty acids (SCFAs) and bile acids that influence host physiology, and immune responses elicited by microbial byproducts like lipopolysaccharides (LPS) reaching the liver and brain. While these interactions are essential for maintaining metabolic homeostasis, microbial dysbiosis can disrupt this equilibrium, promoting systemic inflammation, hepatic insulin resistance, and neuroendocrine dysfunction (61).

Future studies should further elucidate how the gut microbiota shapes neural, endocrine, and metabolic control within this interconnected system. Although SCFAs and bile acids have been identified as major mediators, the vast genetic and functional diversity of the microbiome likely conceals additional mechanisms influencing glucose homeostasis. Integrative approaches combining microbiomics, metabolomics, and neurophysiology will be crucial to bridge this gap (61).

Together, these insights into the gut-liver-brain network underscore how peripheral signals converge on central circuits to regulate glucose metabolism, an integrative framework that extends to the brain-pancreas axis, discussed next.

## 4 The brain-pancreas connection

Extending beyond incretin-mediated pathways, the brain-pancreas axis exemplifies how neuroendocrine circuits integrate peripheral metabolic cues with central control mechanisms to maintain glucose and energy homeostasis. The brain and peripheral nerves coordinate with pancreatic islets to regulate glucose. Glucose-sensing neurons in the hypothalamus, including AgRP/NPY, POMC and VMH, form the core of brain–pancreas connections. These neurons project to the PVN and the brainstem, ultimately communicating with the NTS and DMV. Through these pathways, brainstem structures control vagal and/or spinal outputs to the pancreatic islets, thereby regulating insulin and glucagon secretion (62–64).

Mammals have the capability to maintain blood glucose levels within strikingly narrow ranges under normal conditions. The maintenance of glucose homeostasis relies not only on the intrinsic sensing properties of pancreatic islet cells but also on complex neuroendocrine circuits and autonomic inputs from the sympathetic and parasympathetic nervous systems.

The vagus nerve, a major parasympathetic pathway, further modulates glucose metabolism by influencing both insulin release and hepatic glucose production (65). Direct evidence of autonomic control over islet function was provided by Rodriguez-Díaz et al., who demonstrated in mice with islets transplanted into the eye that autonomic axons innervating the islet directly regulate glucose homeostasis (65).

Pancreatic islets are thus under dual autonomic control. Sympathetic fibers, originating from thoracolumbar spinal segments, stimulate glucagon release from α-cells and suppress insulin secretion from β-cells, largely through modulation of islet blood flow (66).

Parasympathetic vagal input, under hypothalamic regulation, promotes insulin secretion during the cephalic phase of feeding by integrating sensory cues (taste, smell, vision) and responding to gut-derived hormones such as CCK and serotonin. While vagal signaling is dispensable for basal insulin secretion in fasting conditions, it becomes crucial for glucose-stimulated insulin release in the fed state. This dynamic interplay between sympathetic inhibition and parasympathetic stimulation coordinates islet hormone output according to metabolic state (67).

The gut microbiota also influences glucose homeostasis by communicating with the pancreas through various microbial metabolites like the SCFAs and branched-chain amino acids (BCAAs). In both type 1 and type 2 diabetes, microbial imbalance (dysbiosis) alters metabolite profiles, impacting pancreatic islet function. This can lead to effects like increased inflammation, insulin resistance, and the development or progression of diabetes (68). Understanding these complex gut-liver-pancreas-brain circuits could lead to new treatments to prevent or reverse metabolic dysfunction and improve insulin sensitivity.

## 5 Neuroendocrine dysfunction in disease

Neuroendocrine circuits that regulate glucose homeostasis are highly vulnerable to disruption in obesity, type 2 diabetes, and aging. Insulin resistance in the CNS impairs metabolic control, while autonomic dysregulation alters pancreatic function. Reduced vagal tone further limits insulin secretion and weakens counter-regulatory responses to hypoglycemia. Evidence from human and animal studies shows that these disturbances are accompanied by neuroinflammation, neurotransmitter imbalance, and structural brain changes, all of which contribute to progressive metabolic derangements and increased risk of cognitive decline (69).

Hypothalamic inflammation, characterized by gliosis and microglial activation in the ARC nucleus interferes with PI3K-Akt and STAT3 signaling and blunts POMC and AgRP neuronal responsiveness, thereby favoring orexigenic tone and systemic insulin resistance (70, 71).

In leptin-deficient models, chronic inflammatory signaling further exacerbates hyperphagia and metabolic dysfunction. Mechanistic studies implicate mitochondrial dysfunction and endoplasmic reticulum (ER) stress as key contributors, exemplified by POMC-specific Mitofusin 2 (Mfn2) deletion, which induces ER stress, leptin resistance, and obesity (72, 73).

Beyond inflammation, dysregulation of orexigenic neuropeptides such as neuropeptide Y (NPY) and melanin-concentrating hormone (MCH) worsens metabolic disease. In this regard, ablation of NPY Y2 receptors improves glucose and lipid profiles in *ob/ob* mice, whereas MCH deletion increases energy expenditure and glycemic control (74, 75).

Moreover, novel pituitary-brain circuits contribute to systemic glucose regulation, as a subset of AgRP-expressing neurons in the anterior pituitary, which responds to bile acid signals and regulates glucose tolerance independently of food intake; silencing these neurons improves glucose-stimulated insulin secretion (76).

However, the precise mechanisms linking brain activity to peripheral glucose handling in humans remain poorly defined, representing a critical frontier for translational research in metabolic regulation.

## 6 Emerging therapeutic perspectives

The identification of central mechanisms governing glucose homeostasis has opened new therapeutic avenues for obesity, insulin resistance, and diabetes. For instance, intranasal insulin bypasses the blood-brain barrier to enhance central insulin action, improving hepatic glucose production and peripheral glucose disposal without raising systemic insulin levels (77).

Dual and triple incretin receptor agonists, such as tirzepatide and retatrutide, combine peripheral and central actions that target mechanisms underlying sex differences in metabolic homeostasis and disease. These agents not only improve glycemic control and promote weight loss but also partially restore leptin sensitivity through microbiota-derived metabolites such as inosine (78, 79).

Systematically incorporating sex as a biological variable in both preclinical and clinical research will be essential to uncovering mechanistic differences, improving translational accuracy and ultimately guiding the development of sex-adapted therapies for diabetes and other metabolic disorders (80).

Neuromodulatory strategies, including vagus nerve stimulation, selective hepatic-celiac vagal modulation, optogenetics, and chemogenetics, also show promise in improving glycemic control and reducing inflammation (81, 82).

Lifestyle interventions, including intermittent fasting and time-restricted feeding, engage hypothalamic and autonomic pathways to reduce hepatic gluconeogenesis and enhance metabolic flexibility, while early-life nutritional modulation can shape hypothalamic plasticity and long-term glucose control (83).

Emerging therapies targeting mitochondrial quality control, inflammatory signaling, and post-translational modifications (e.g., neddylation) also hold potential to restore central metabolic function. Moreover, multi-omics approaches, integrating proteomics, metabolomics, epigenomics, and microbiomics, combined with artificial intelligence and circuit-level mapping, are accelerating biomarker discovery and precision therapeutic design (84).

Non-invasive neuroimaging tools, including positron emission tomography (PET) and functional magnetic resonance imaging (fMRI), complement these approaches by enabling longitudinal monitoring of brain metabolism and activity in humans (85–87).

## 7 Discussion

Central regulation of glucose homeostasis arises from the coordinated activity of neuroendocrine and autonomic circuits integrating hormonal and nutrient-derived signals from adipose tissue, gut, liver, and pancreas. Within this network, leptin, insulin, and incretin pathways converge on AgRP/NPY and POMC neurons in the ARC to control feeding, hepatic glucose output, and insulin sensitivity (4–14).

These hypothalamic circuits are developmentally programmed yet remain modifiable during early life. Maternal obesity, overnutrition, or inflammation can durably alter AgRP and POMC connectivity through microglial activation and impaired perineuronal net (PNN) maturation, establishing a “metabolic memory” that predisposes offspring to lifelong glucose dysregulation (29–35).

The brainstem complements hypothalamic control by integrating visceral feedback within the gut-liver-brain axis, central to metabolic homeostasis and a target for novel therapies (60–64). Chronic overnutrition and neuroinflammation exacerbate these impairments, promoting hypothalamic leptin and insulin resistance, hallmarks of metabolic disease and contributors to cognitive decline (70, 71).

Next-generation incretin mimetics and neuromodulatory interventions capitalize on these central-peripheral interactions to enhance metabolic flexibility and restore hormonal sensitivity (78, 79, 81–83). Integrating multi-omics and high-resolution neurocircuit mapping will refine biomarker discovery, improve translational fidelity, and inform precision strategies linking metabolic, neuroendocrine, and immune systems (84).

In summary, glucose homeostasis is governed by a distributed, plastic neuroendocrine network whose developmental and inflammatory plasticity shapes the trajectory of metabolic health. Leveraging this integrative understanding, bridging developmental neurobiology, incretin pharmacology, and neuromodulation, will be key to achieving durable metabolic restoration in obesity and diabetes.

## 8 Limitations and future directions

Despite major advances, translating mechanistic insights from animal models to humans remains a key challenge. Standardization across strains, diets, and experimental designs is needed to improve reproducibility and cross-study comparability. Longitudinal human cohorts combining neuroimaging, biofluid omics, and hormonal profiling will be instrumental to map central-peripheral interactions in metabolic control.

Future studies should incorporate sex as a biological variable, as estrogens acting through ERα signaling improve insulin sensitivity, β-cell survival, and central metabolic regulation, providing protection before menopause. Developing biomarkers to monitor central interventions will also accelerate translation into effective therapies.
